# Effects of different X-ray irradiation dosages on *Wolbachia* trans-infected and uninfected *Aedes aegypti* mosquitoes

**DOI:** 10.1371/journal.pntd.0014563

**Published:** 2026-08-28

**Authors:** Jiraporn Yongyai, Parinda Thayanukul, Pattamaporn Kittayapong

**Affiliations:** 1 Center of Excellence for Vectors and Vector-Borne Diseases, Faculty of Science, Mahidol University, Salaya, Nakhon Pathom, Thailand; 2 Department of Biology, Faculty of Science, Mahidol University, Bangkok, Thailand; 3 EcoHealth Research Center, Go Green Co., Ltd., Chachoengsao, Thailand; University of Queensland - Saint Lucia Campus: The University of Queensland, AUSTRALIA

## Abstract

**Background:**

*Aedes aegypti* is a primary vector of several arboviral diseases, including dengue, chikungunya, Zika, and yellow fever. Eliminating *Ae. aegypti* can disrupt virus transmission cycles, thereby reducing the incidence of these diseases. Releasing sterile *Ae. aegypti* males has been implemented to suppress their natural populations, using three approaches, i.e., ionizing radiation-induced sterilization (Sterile Insect Technique, SIT), *Wolbachia*-induced cytoplasmic incompatibility (Incompatible Insect Technique, IIT), and a combination of both approaches (SIT/IIT).

**Methodology/principal findings:**

This study investigated effects of different X-ray radiation doses on the ecological fitness of the Thai wild-type and *Wolbachia* trans-infected *Ae. aegypti*. Both wild-type and *w*AlbB trans-infected *Ae. aegypti* were irradiated with different X-ray radiation dosages, i.e., 30, 50, and 70 Gy, at the pupal stage. Our results demonstrated reduced fitness parameters in both *Ae. aegypti* strains when irradiation dosages increased, with greater adverse effects observed in the *Wolbachia-*infected group. Interestingly, 30 Gy irradiation appeared to prolong the lifespan of *w*AlbB trans-infected *Ae. aegypti*. Male fertility in both wild-type and *w*AlbB trans-infected mosquitoes irradiated at 50 Gy and 70 Gy was nearly zero. *Wolbachia* density generally declined with increasing radiation, and higher doses were associated with reduced mating competitiveness. At 30 Gy, wild-type males outperformed *w*AlbB trans-infected males in mating competitiveness; but it was reversed at 50 Gy and 70 Gy.

**Conclusions/significance:**

Based on the evaluation of ecological fitness parameters and mating competitiveness across various X-ray dosages under laboratory conditions, 30 Gy is recommended for sterilizing both Thai wild-type and *w*AlbB trans-infected *Ae. aegypti*. Our findings aim to improve the efficiency of sterile mosquito release programs, thereby suppressing *Ae. aegypti* vector populations and mitigating the risk of arboviral disease transmission*.*

## Author summary

Mosquito-borne diseases, especially dengue, chikungunya, Zika and yellow fever are still public health concerns. Controlling *Aedes aegypti* vector could reduce these diseases. The release of sterile mosquitoes, using Sterile Insect Technique (SIT) or SIT combined with Incompatible Insect Technique (IIT), becomes popular approach to control mosquito vectors. In this study, we investigated the effect of various X-ray irradiation dosages on adult emergence, survival, sterility, and mating competitiveness of both wild-type and *Wolbachia*-infected *Ae. aegypti* mosquitoes. In addition, we assessed cytoplasmic incompatibility (CI) and *Wolbachia* density of irradiated *Wolbachia*-infected *Ae. aegypti*. Our findings indicated that X-ray radiation between 30 Gy to 70 Gy had no observable effect on adult emergence of wild-type *Ae. aegypti*, but slightly reduced emergence of *Wolbachia-*infected ones. Increasing X-ray doses reduced survival of wild-type mosquitoes, whereas *Wolbachia*-infected ones exhibited a slight increase in survival at 30 Gy. In CI tests, females still laid eggs but CI crosses consistently yielded zero larvae. All irradiated *Wolbachia*-infected *Ae. aegypti* groups exhibited a reduction in *Wolbachia* density when compared to non-irradiated control. Irradiated females could maintain higher *Wolbachia* density than irradiated males. For mating competitiveness, high radiation reduced competitiveness in wild-type males and *w*AlbB-infected *Ae. aegypti*. In conclusion, an X-ray dose of 30 Gy was suggested for sterilizing both wild-type and *Wolbachia*-infected *Ae. aegypti* mosquitoes before an application in SIT or combined SIT/IIT programs in order to control *Ae. aegypti* mosquito populations.

## Introduction

*Aedes aegypti* is a primary vector of several arboviral diseases, including dengue, chikungunya, Zika, and yellow fever*.* This species is found in tropical and subtropical regions across Asia, Africa, Europe, the Americas, and Oceania [1]. It typically inhabits artificial containers or natural water reservoirs near human dwellings, both indoors and outdoors, in urban and rural settings [2]. Eliminating *Ae. aegypti* can disrupt the viral transmission cycle and reduce the incidence of associated diseases [3].

Various strategies have been employed to control *Ae. aegypti* population, encompassing environmental, chemical, and biological approaches [4]. Environmental control measures, such as covering or emptying water-filled containers, are widely practiced [5]. Chemical control involves the use of insecticides, but many mosquito species have developed resistance to these chemicals [6], and their application can harm non-target organisms. In contrast, biological control, including the release of biological agents or sterile mosquitoes, is more environmentally sustainable [7]. Recently, the Sterile Insect Technique (SIT) and Incompatible Insect Technique (IIT) have gained attention, SIT involves releasing radiation-sterilized males, while IIT uses *Wolbachia*-infected males that induce reproductive incompatibility. A combined SIT/IIT strategy has also been implemented in many countries to harness the advantages of both methods for suppressing *Ae. aegypti* populations, such as in Yucatan, Mexico [8]; Singapore [9]; and Chachoengsao, Thailand [10].

Ionizing radiation induces sterility in male mosquitoes by causing chromosomal damage or lethal mutations in sperm [11]. Incomplete sperm development leads to embryonic mortality. However, increased irradiation doses can reduce mating competitiveness—a critical factor for SIT success [12]. In addition, irradiation reduced adult longevity and transiently delayed sexual maturation and insemination capacity [13,14]. In *Wolbachia*-trans-infected mosquitoes, irradiation also caused ovary-specific depletion of *Wolbachia* titers, whereas no reduction was observed in somatic cells [15]. Moreover, irradiation at early pupal stages further exacerbated the decline in *Wolbachia* density, potentially compromising cytoplasmic incompatibility and pathogen interference effects [15]. While high irradiation doses are effective for sterilization, they often compromise fitness, whereas lower doses may fail to ensure complete sterility.

Gamma rays from cobalt-60 (Co–60) are commonly used for mosquito sterilization [16,17], but handling and disposing of radioactive waste pose significant challenges [18]. Radioactive isotope materials require stringent security and regulatory controls and entail high investment costs. Consequently, X-ray has been proposed as an alternative. X-rays are electrically generated by the electron beams strike high atomic number materials, producing Bremsstrahlung radiation [19]. Unlike gamma rays from radioisotopes, which have discrete energy levels, Bremsstrahlung exhibits a broad energy spectrum. Under specific irradiator configurations and controlled operational conditions, the penetration depth, dose uniformity, and biological effects for *Ae. aegypti* sterilizations of X-rays were comparable to those of Co-60 gamma radiation [19–21]. Therefore, X-rays present a promising alternative to gamma rays from cobalt-60.

*Wolbachia* is an intracellular bacterium naturally present in mosquito species such as *Armigeres subalbatus, Aedes albopictu*s*,* and *Culex pipiens*, but not typically in wild *Ae. aegypti* [22]. Recent studies have found *Wolbachia* in natural populations of *Ae. aegypti*, despite previous beliefs that this mosquito species did not naturally harbor the *Wolbachia* [23,24]. However, several groups have successfully microinjected *Wolbachia* into *Ae. aegypti* establishing stably infected lines for biological control [25]. This approach exploits cytoplasmic incompatibility (CI), whereby *Wolbachia*-infected males mating with uninfected females produce inviable embryos [22,26]. *Wolbachia* also inhibits arbovirus replication in mosquitoes, lowering disease transmission risk [27].

*Wolbachia*-based approach generally follows a replacement model, releasing both sexes into the field. In contrast, SIT and combined SIT/IIT rely on the suppression model, releasing only males. While the replacement strategy faces criticism for potentially increasing nuisance mosquito populations, the suppression approach is constrained by the cost and complexity of sex separation and mass-rearing. In addition, *Wolbachia* infection might be lost in the environment, especially under high-temperature field conditions [28], causing unsuccessful implementation.

Combining SIT and IIT offers a synergistic solution. *Wolbachia* provides a second safeguard in cases of incomplete sterilization, allowing for lower irradiation doses. Moreover, if *Wolbachia*-infected females are accidentally released, they are less likely to transmit viruses due to the symbiont’s antiviral properties. Females are also more radiosensitive than males, typically failing to produce viable eggs post-irradiation [16], reducing the risk of *Wolbachia* establishment in the field of suppression model.

A prior study irradiated Thai *Wolbachia* trans-infected *Ae. aegypti* with 50 Gy and 70 Gy of gamma rays by Cobalt-60 to determine the optimal doses for use in a combined SIT/IIT strategy [17]. This led to a field trial employing 70 Gy gamma irradiation in Thailand [10]. Nevertheless, the effects of different X-ray irradiation doses on Thai *w*AlbB trans-infected *Ae. aegypti*, particularly with respect to ecological fitness, remains insufficiently characterized for application in a combined SIT/IIT program.

This study aimed to evaluate the effects of various X-ray doses on fitness parameters including, survival, longevity, male sterility, and male mating competitiveness of both wild-type and *w*AlbB trans-infected *Ae. aegypti* mosquitoes*.* We also addressed the impact on CI mating and *Wolbachia* density in irradiated mosquitoes. These findings would support improvement in sterile mosquito release programs and help mitigate arboviral disease transmission risks*.*

## Materials and methods

### Mosquito colony and rearing

The wild-type *Aedes aegypti* colony (Aae-JJ) originated from the eggs collected using ovitraps in Bangkok, Thailand. The Thai *w*AlbB trans-infected *Ae. aegypti* colony (*w*AlbB-TH) was developed by trans-infecting the *w*AlbB strain from *Ae. albopictus* into the Aae-JJ colony via adult stage microinjection, as previously described [29]. Both colonies were maintained under standard insectary conditions at 27.0°C ± 2.0°C, 70.0% ± 5.0% relative humidity, and a 12:12 light-dark cycle. Eggs were hatched in screw-cap glass containers using cooled, boiled-filtered water. Larvae were reared in plastic trays (30 cm × 40 cm × 5 cm) containing 2 L of dechlorinated filtered water, at a density ~0.25 larvae/mL (~500 larvae/tray). They were fed ad libitum with a diet of fish meal (Chanpongcharoen Kankaset Supplier, Thailand), crushed chicken liver, and yeast powder (Cheese Powder Supplier, Thailand) in a ratio of 3.3: 0.1: 0.56 by weight. Feeding ceased at the pupal stage. Pupae were transferred to cages sized 30 cm × 30 cm × 30 cm, and provided with 10% sucrose-soaked cotton in a cotton-filled container.

### Pupal irradiation

Male and female pupae were separated using a locally modified pupal sex separator from the Model 5412 (John Hock Co., Ltd, USA). Approximately 300 pupae were placed in a transparent plastic container (diameter: 12.5 cm, height: 14.5 cm) with 300 mL of dechlorinated filtered water and transported to the irradiation facility. The pupae, aged 6–12 hours post-pupation, were irradiated. Three X-ray doses, 30 Gy (15.0 Gy/min, 120 s), 50 Gy (14.3 Gy/min, 210 s), and 70 Gy (14.0 Gy/min, 300 s) were applied using an RS 2400•Q Irradiator (Rad Source Technologies Inc., USA) at the Thailand Institute of Nuclear Technology (TINT). Non-irradiated pupae (0 Gy) served as controls.

### Adult emergence and survival

For adult emergence in each experimental group, 100 male or 100 female pupae were separated into different cages (20 cm × 20 cm × 20 cm). Pupae were placed in an open Petri dish for emergence. Each treatment had three replicates. Adults were provided with 10% sucrose solution. The number of emerging adults was counted on the first day of emergence. Emergence rate (%) was calculated as (Number of adults/ Number of pupae) × 100.

For the adult survival test, 30 male and 30 female adults from each colony at different dose groups were placed in separate cages, with three replicates per treatment. All mosquitoes were provided with 10% sucrose solution. Daily mortality was recorded until all individuals had died.

### Male sterility and cytoplasmic incompatibility (CI) tests

Sterility tests were conducted between irradiated males (wild-type or *w*AlbB trans-infected) and non-irradiated females from the same colonies. CI tests were performed by mating irradiated *w*AlbB trans-infected males with non-irradiated wild-type females. In each replicate, 30 males and 30 females were caged together for one week (four replicates per group) and warm pig blood was offered to females on days 4–6. On day 7, each female was transferred to an individual oviposition container. Eggs were counted under a stereomicroscope. Eggs were hatched in dechlorinated filter water and assessed at the second instar larval stage.

### Male mating competitiveness and induced sterility assessment

Two experimental groups were established based on the sterile male types (irradiated wild-type or *w*AlbB trans-infected), each tested at three mating ratios with non-irradiated wild-type females; i.e., 50:50:50 (non-irradiated wild-type males, sterile males, and non-irradiated wild-type females) – Ho1, 50:250:50 – Ho5, and 50:500:50 – Ho10, following Zhang et al. (2016) [30]. Three X-ray doses (30 Gy, 50 Gy, and 70 Gy) were tested. Each treatment had three replicates, consistent with the methodology of many studies reviewed by Bouyer et al. (2020) [31]. Two controls were included: Hn – non-irradiated wild-type males mated with non-irradiated wild-type females (normal) and Hs – sterile males mated with non-irradiated wild-type females (sterile control). Sterile and fertile males were introduced into the 30 cm × 30 cm × 30 cm cage one hour before female introduction. Blood feeding occurred on days 4–6, and females were then isolated for oviposition. Egg counts and hatch rates were recorded**.** The Fried competitiveness index (C-value) was calculated as C = [(Hn - Ho)/ (Ho - Hs)] × (N/S) [32]. Where Hn was the hatch rate of fertile pairs; Hs was the hatch rate of the cross between fertile females and sterile males; Ho was the hatch rate in the observed tests; N was the number of fertile males; and S was the number of sterile males. Induced sterility (% IS) was calculated as IS (%) = [1 - (number of larvae/number of eggs)] × 100 [33]. Competitiveness was calculated based on en masse egg collection [34]. Hatch rate was defined as the number of hatched larvae (L1-2) divided by the total number of eggs counted per cage.

### *Wolbachia* density

*Wolbachia* density was measured in male and female *w*AlbB trans-infected *Ae. aegypti* that were exposed to 0 Gy, 30 Gy, 50 Gy, or 70 Gy at 7, 14, and 21 days post-emergence with six biological replications. DNA was extracted from whole individual bodies using the DNeasy Blood & Tissue Kit (Qiagen, Germany). Each qPCR reaction contained 2 μl of 1:10 diluted DNA extract, 5 μl SYBR iTaq Universal Green Supermix (BIO-RAD, USA), 0.5 μl of each primer, and 2 μl of DEPC water (Invitrogen, USA). A qPCR was performed using the following program: 95°C for 3 min, 40 cycles of 95°C for 5 s, 59°C for 30 s, and 74.5°C for 10 s. The *wsp* gene (*Wolbachia* surface protein) primers were WSP_F (GCATCTTTTATAGCTGGTGG) and WSP_R (GGAGTGATAGGCATATCTTTCAAT) [27,35]. The *hth* (host homothorax) gene primers were HTH_F (TGGTCCTATATTGGCGAGCTA) and HTH_R (TCGTTTTTGCAAGAAGGTCA) [36]. The cycle threshold (Ct) values of the *Wolbachia* surface protein gene (target) and the *homothorax* gene (reference) were recorded for each sample. Relative *Wolbachia* density was calculated using the 2^ΔCt^ method, where ΔCt = Ct(reference) - Ct(target). The *Wolbachia* density was then expressed as 2^ΔCt^ [37,38].

### Data analysis

All statistical analyses were performed using SPSS version 30.0 (IBM Corp., USA), Microsoft Excel (Microsoft Corp., USA), and R version 4.5.2 (Posit Software, USA). Data normality was assessed using the Shapiro-Wilk test. When assumptions of normality were not met, non-parametric tests were applied.

Differences in adult emergence, male sterility, and cytoplasmic incompatibility (CI) among irradiation treatments were analyzed using the Kruskal-Wallis test, followed by Dunn’s post hoc pairwise comparisons. Comparisons between sex or colony effects for these parameters were conducted using the Mann–Whitney U test.

Adult survival was analyzed using the Kaplan-Meier method to estimate median survival times, implemented with the survival package (version 3.7.0) in R [39]. Differences among survival curves across irradiation treatments were assessed using pairwise log-rank tests with Benjamini–Hochberg correction for multiple comparisons.

*Wolbachia* density was analyzed using a linear mixed-effects model implemented in the afex package (version 1.5-1) [40], with mosquito age and irradiation treatment as fixed effects, and cage (repeat) included as a random effect. The inclusion of cage as a random effect accounts for the non-independence of observations due to repeated sampling of individuals from the same experimental cage across time points. Tukey’s post hoc tests were applied for multiple comparisons.

Data from the mating competitiveness experiments, including egg hatch rate, induced sterility percentage, and Fried competitiveness index (C index), were tested for normality using the Shapiro-Wilk test. Homogeneity of variances was assessed using Levene’s test. Comparisons between mosquito colonies were performed using independent-samples *t*-tests when the assumption of equal variances was met; otherwise, Welch’s *t*-test was applied. For comparisons among irradiation or mating-ratio treatments, one-way analysis of variance (ANOVA) was used when the assumptions of normality and homogeneity of variances were met. When these assumptions were violated, non-parametric analysis was conducted using the Kruskal-Wallis test, followed by Dunn’s post hoc pairwise comparisons.

## Results

### Adult emergence

**Fig 1:**
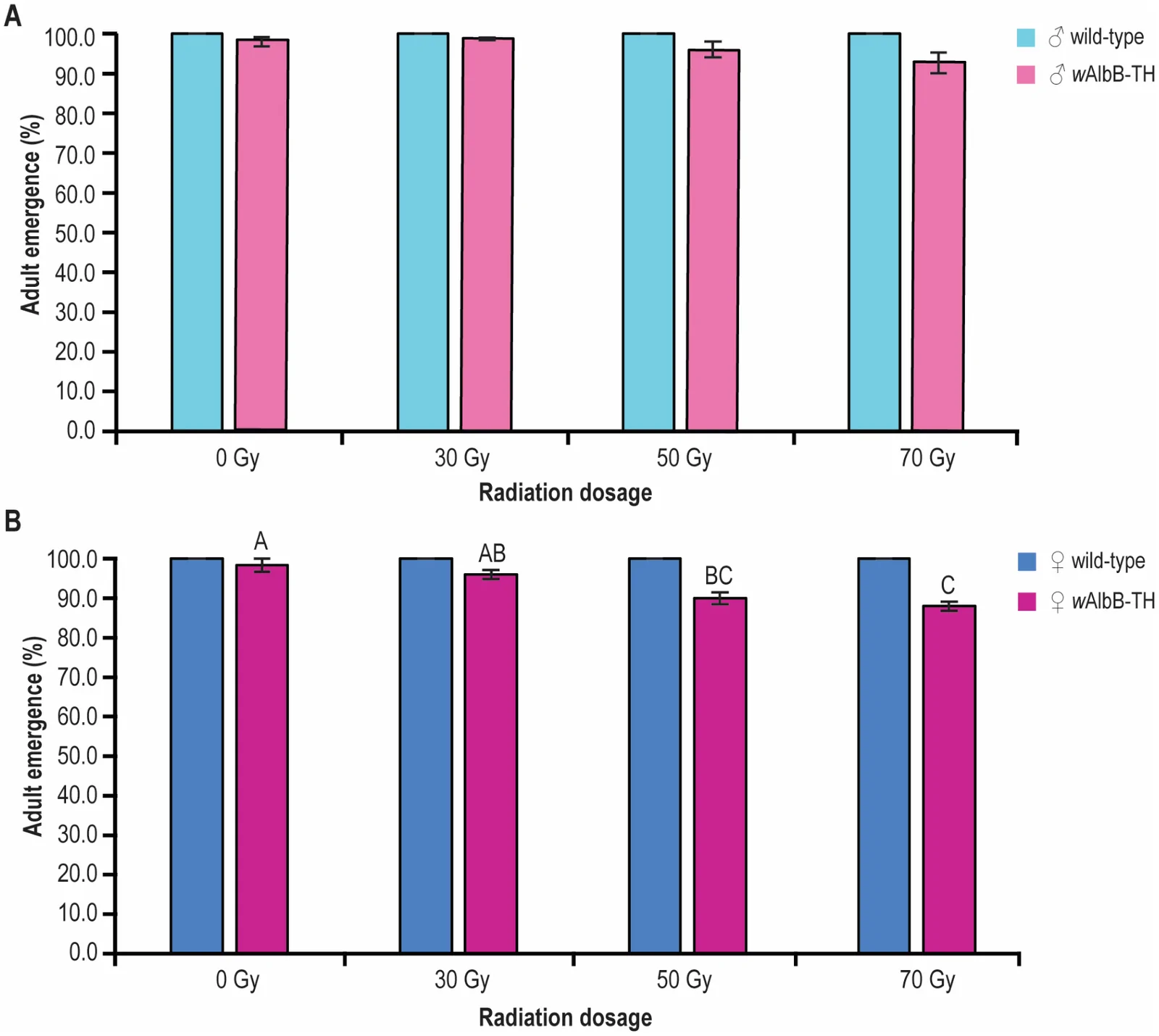
Adult emergence percentages of (A) males and (B) females wild-type *Aedes aegypti* and *w*AlbB trans-infected *Ae. aegypti* following X-ray irradiation at 30 Gy, 50 Gy, and 70 Gy, along with a non-irradiated control (0 Gy). Bars sharing identical or no letters indicate no significant difference among radiation treatments (*p* > 0.05). Error bars represent the standard error (SE) from three replicates of 100 larvae each.

The adult emergence rates of both male and female wild-type *Ae. aegypti* mosquitoes were 100% in both non-irradiated and all irradiated treatments (Fig 1). However, irradiated *w*AlbB trans-infected *Ae. aegypti* males exhibited emergence rates ranging from 93% to 99% (Fig 1A).

For *w*AlbB trans-infected females, emergence rates ranged from 88% to 98% across irradiated treatments, while emergence rates of wild-type *Ae. aegypti* was 100% in all irradiated treatment (Fig 1B). In the 50 Gy and 70 Gy irradiated *w*AlbB trans-infected female groups, emergence was significantly lower than that in the non-irradiated controls (Kruskal-Wallis test, *df* = 3, *p* = 0.030).

Overall, irradiation between 30 Gy–70 Gy had no observable effect on adult emergence in wild-type *Ae. aegypti*, but slightly reduced emergence in *w*AlbB trans-infected *Ae. aegypti*.

### Adult survival rate

**Fig 2:**
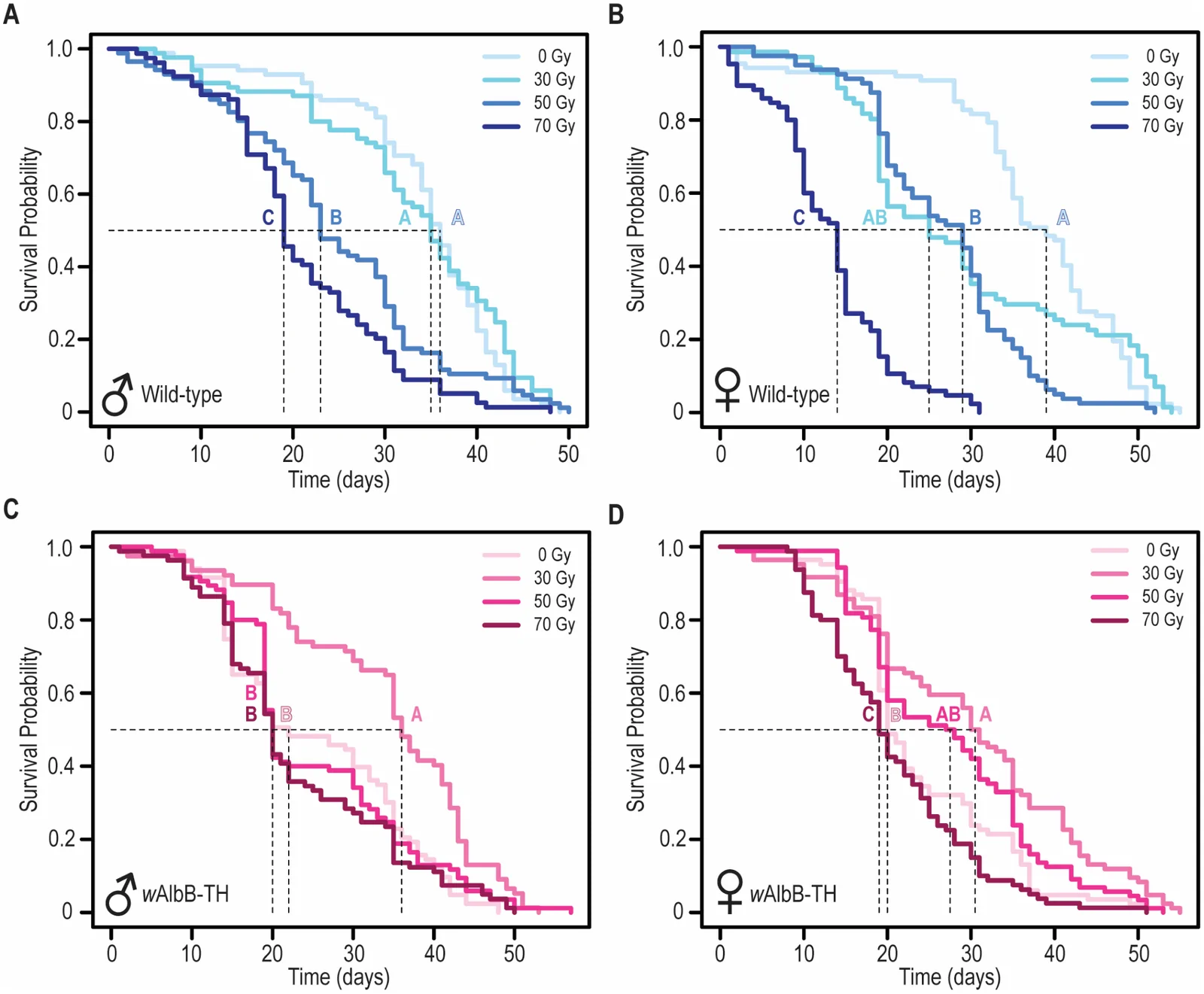
Survival rates of (A) male and (B) female wild-type *Aedes aegypti*, and (C) male and (D) female *w*AlbB trans-infected *Ae. aegypti* following X-ray irradiation at 30 Gy, 50 Gy, and 70 Gy, along with a non-irradiated control (0 Gy). Data were derived from three replicated sets of 30 adults. Identical letters indicate no significant difference (*p* > 0.05).

Wild-type *Aedes aegypti* males exposed to 50 Gy and 70 Gy exhibited significantly reduced survival rates when compared to the control and 30 Gy treatments (Log-rank test, *df* = 3, χ2 = 77.4, *p* < 0.001) (Fig 2A and S1 Table). However, in wild-type *Ae. aegypti* females, all irradiation treatments (30 Gy–70 Gy) adversely affected survival, with similar reduction patterns observed at 30 Gy and 50 Gy (Fig 2B). The survival at 50 Gy and 70 Gy was significantly lower than the control (Log-rank test, *df* = 3, χ2 = 226, *p* < 0.001).

Interestingly, 30 Gy-irradiated *w*AlbB trans-infected males and females had significantly higher survival rates than non-irradiated controls (Log-rank test, ♂ *w*AlbB-TH: *df* = 3, χ2 = 30.7, *p* < 0.001; ♀ *w*AlbB-TH: *df* = 3, χ2 = 37.1, *p* < 0.001) (Fig 2C–2D and S1 Table). For both sexes, survival in the 50 Gy treatment was similar to controls, but 70 Gy-treated females showed slightly reduced survival.

These results suggest that wild-type mosquitoes were more susceptible to higher X-ray radiation doses, whereas *w*AlbB-trans-infected mosquitoes exhibited minimal change overall and even increased survival at 30 Gy.

### Male sterility and CI tests

**Fig 3:**
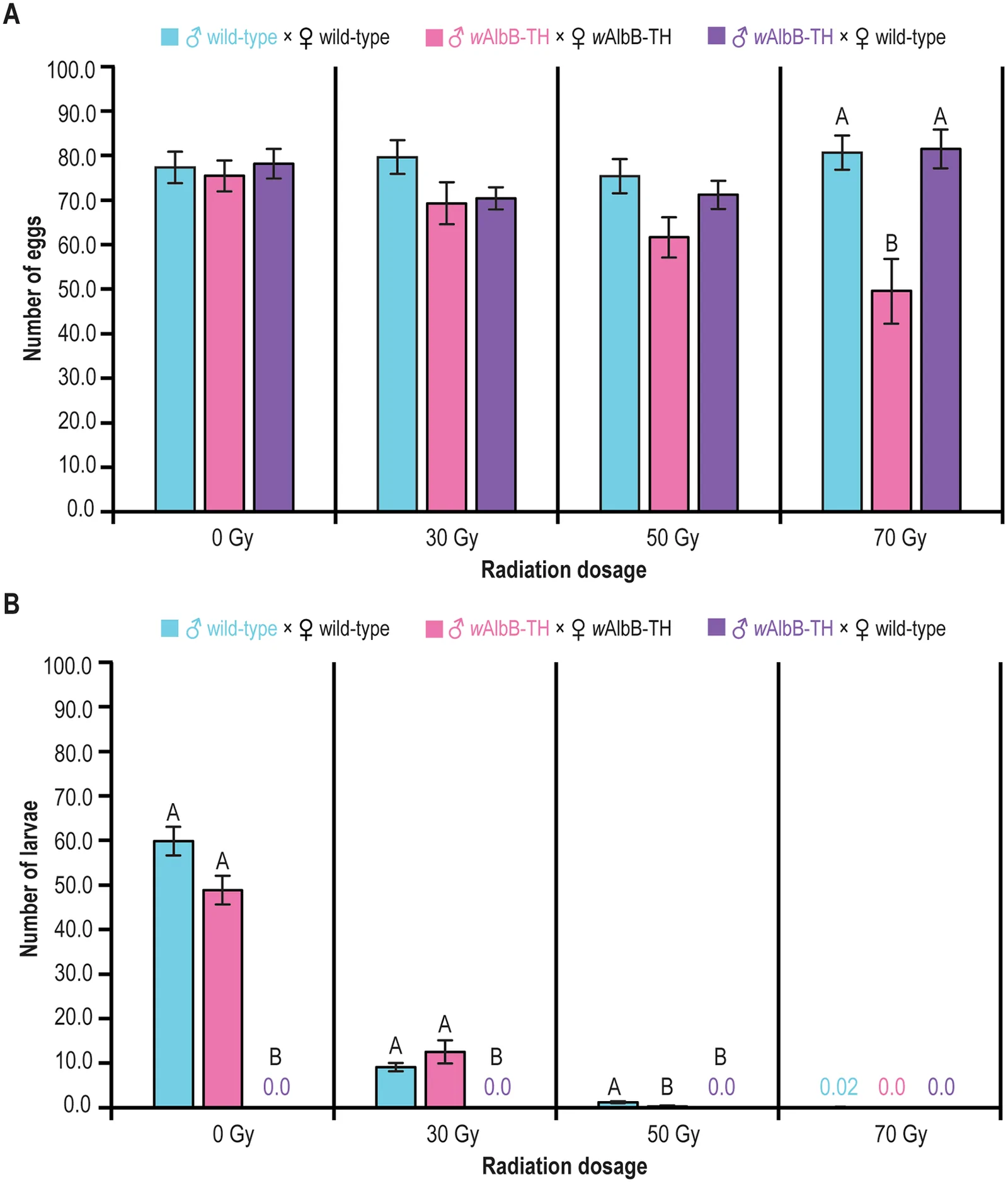
Egg (A) and larval (B) counts from male sterility and cytoplasmic incompatibility tests. Males were irradiated at 30 Gy, 50 Gy, or 70 Gy, except for the control set (0 Gy), **and mated with non-irradiated females.** Bars sharing identical letters denote no statistically significant difference among the male treatment group (*p* > 0.05). Error bars represent standard error (SE) from four replicates.

Irradiated and non-irradiated wild-type *Ae. aegypti* male mating tests showed numbers of eggs in an average range of 75–81. The number of eggs among three mating groups were not statistically different in non-irradiated (Kruskal-Wallis test, *df* = 2, *p* = 0.631), 30 Gy-irradiated (Kruskal-Wallis test, *df* = 2, *p* = 0.066), and 50 Gy-irradiated (Kruskal-Wallis test, *df* = 2, *p* = 0.067) male mating tests (Fig 3A). However, at 70 Gy, egg numbers from *w*AlbB trans-infected males mated with infected females were significantly lower than other combinations (Kruskal-Wallis test, *df* = 2, *p* = 0.002). Egg production in the mating group of *w*AlbB trans-infected males and females decreased with increasing radiation dosage (Kruskal-Wallis test, *df* = 3, *p* = 0.016), whereas egg production in the mating group of wild-type male and female remained consistent (Kruskal-Wallis test, *df* = 3, *p* = 0.630).

Larval hatching was completely absent in all crosses involving *w*AlbB trans-infected males and wild-type females due to cytoplasmic incompatibility (Fig 3B). Larval counts from wild-type male crosses were significantly higher than *w*AlbB trans-infected crosses at 50 Gy (Kruskal-Wallis test, *df* = 2, *p* < 0.001). At 70 Gy, larval numbers in all groups were near zero.

In conclusion, females laid eggs in all treatments, although fewer eggs were produced in *w*AlbB trans-infected groups at high radiation doses. On the other hand, larval emergence was drastically reduced for all irradiated groups. CI cross consistently yielded zero larvae.

### *Wolbachia* density

**Fig 4:**
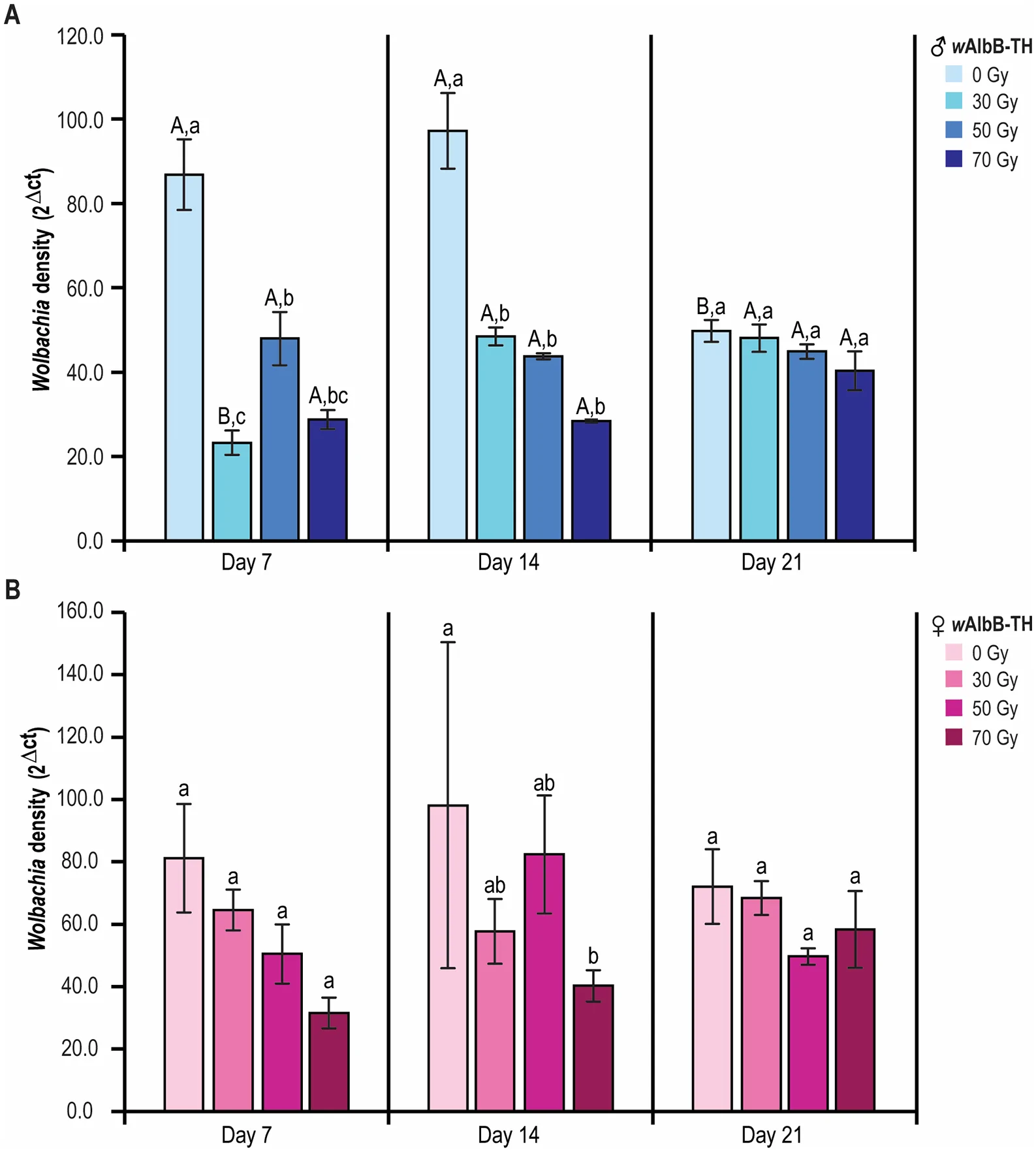
Relative *Wolbachia* density of *w*AlbB trans-infected *Aedes aegypti* males (A) and females (B) measured at 7, 14, and 21 days post-emergence following pupal irradiation at 30, 50, and 70 Gy, with a non-irradiated control. For bars indicated with identical upper-case letters, the *Wolbachia* density does not differ significantly between different mosquito ages within each radiation dose (*p* > 0.05). For bars indicated with identical lower-case letters, the *Wolbachia* density does not differ significantly between radiation dose within mosquito age (*p* > 0.05). Vertical error bars indicate standard error (SE) from the six biological replications.

In male groups treated with 30 Gy, *Wolbachia* levels increased significantly over time (30 Gy; day 7 = 23.26 ± 2.93, day 14 = 48.50 ± 2.09, and day 21 = 48.11 ± 3.24), whereas those in the 50 Gy and 70 Gy groups remained relatively stable (Fig 4A). Conversely, *Wolbachia* levels decreased significantly by day 21 in aged, non-irradiated male mosquitoes (day 7 = 86.82 ± 8.35, day 14 = 97.23 ± 8.94, and day 21 = 49.81 ± 2.82). The mean *Wolbachia* levels were higher in non-irradiated males compared to irradiated males across all age groups, though it was not statistically significant at day 21, indicating that radiation negatively affected *Wolbachia* density. A linear mixed-effects model indicated that both age and radiation treatment in male mosquitoes had an interactive effect on *Wolbachia* density (*F*_*6,55*_ = 14.942, *p* < 0.001)*.*

In female, *Wolbachia* levels in the 30 Gy group remained relatively stable from days 7–21 (30 Gy; day 7 = 64.52 ± 6.55, day 14 = 57.68 ± 10.38, and day 21 = 68.44 ± 5.47) (Fig 4B). In non-irradiated (0 Gy; day 7 = 81.20 ± 17.41, day 14 = 98.13 ± 52.27, and day 21 = 72.09 ± 11.95) and 50 Gy-treated (50 Gy; day 7 = 50.50 ± 9.53, day 14 = 82.42 ± 18.92, and day 21 = 49.69 ± 2.61) females, *Wolbachia* densities peaked on day 14 before returning to the prior levels by Day 21. Similar to males, *Wolbachia* densities in irradiated females were lower than those in non-irradiated controls across all ages, although it was not statistically significant. Radiation treatment had a significant impact on *Wolbachia* density in female mosquitoes (*F*_*3,55*_ = 4.997, *p* < 0.01).

All irradiated *w*AlbB trans-infected *Ae. aegypti* groups (both male and female) exhibited lower relative *Wolbachia* abundance compared to non-irradiated ones. Radiation treatment reduced *Wolbachia* density relative to non-irradiated controls. While increasing radiation dosages appeared to exert a stronger influence on *Wolbachia* density, although this relationship was not statistically significant.

### Male mating competitiveness

**Fig 5:**
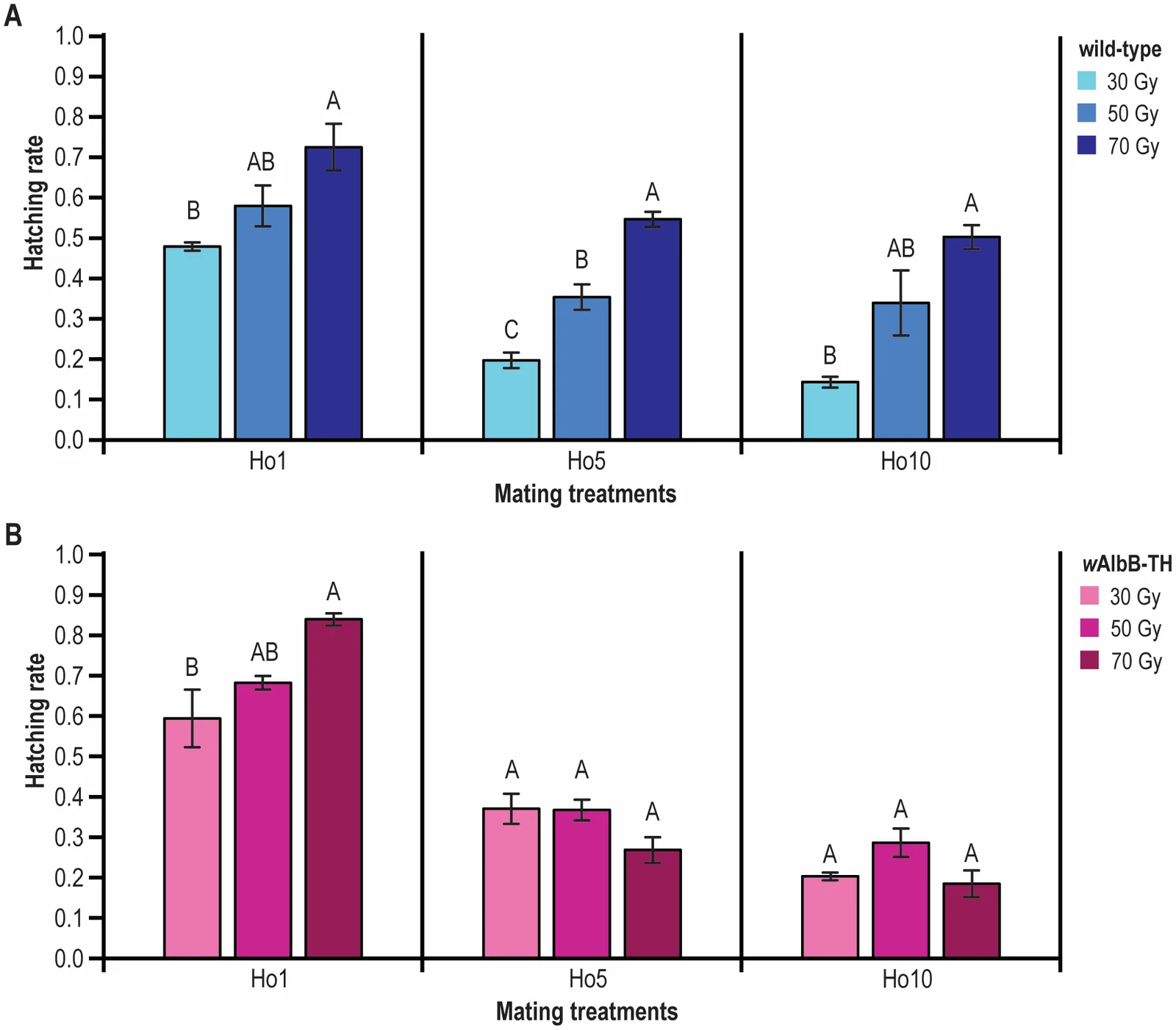
Egg hatching rates from mating competitiveness tests of wild-type (A) and *w*AlbB trans-infected (B) *Aedes aegypti* with males being irradiated at 30 Gy, 50 Gy, or 70 Gy. Mating ratios of fertile males: sterile males were Ho1 (1:1), Ho5 (1:5), and Ho10 (1:10). Bars with identical letters or letters indicate no significant difference between radiation doses in each treatment (*p* > 0.05). Error bars show SE from three replicates.

In male mating competitiveness study, egg hatch rates decreased as the ratio of sterile males increased (Ho1, Ho5, Ho10) (Fig 5A and 5B). For wild-type groups, 30 Gy treatments produced significantly lower hatch rates than 50 Gy and 70 Gy across all ratios (Ho1: *F*_*2,6*_ = 7.570, *p* = 0.023; Ho5: *F*_*2,6*_ = 50.609; *p* < 0.001; Ho10: *F*_*2,6*_ = 12.789, *p* = 0.007, respectively).

This trend was mirrored in *w*AlbB trans-infected *Ae. aegypti* male groups at Ho1. In Ho1, the hatch rates of *w*AlbB trans-infected mosquitoes were lower in 30 Gy (0.59 ± 0.07) and 50 Gy (0.68 ± 0.02) treatments when compared to those of 70 Gy (0.84 ± 0.02) treatments (Kruskal-Wallis: Ho1; *df* = 2, *p* = 0.038). No significant difference was observed in Ho5 (Ho5: *F*_*2,6*_ = 3.371, *p* = 0.104) and Ho10 groups (Ho10: *F*_*2,6*_ = 3.229, *p* = 0.112).

The hatch rate of 30 Gy-treated *w*AlbB trans-infected male group was not significantly different from that of 30 Gy-treated wild-type group in Ho1 (Welch’s *t*-test, *p* = 0.251) (S1A Fig). This trend was also observed for the Ho1 groups in other treatment doses (50 Gy: Independent *t*-test, *p* = 0.115, 70 Gy: Independent *t*-test, *p* = 0.128). However, the 30 Gy-treated *w*AlbB trans-infected male groups in Ho5 (Independent *t*-test, *p* = 0.017) and Ho10 (Independent *t*-test, *p* = 0.024) ratios produced higher hatch rates than those of the wild-type group (S1A Fig). In contrast, 70 Gy-treated *w*AlbB trans-infected male group had lower hatch rates than wild-type sterile male mating groups in Ho5 (Independent *t*-test, *p* = 0.001) and Ho10 (Independent *t*-test, *p* = 0.002) observed ratios (S1C Fig).

**Fig 6:**
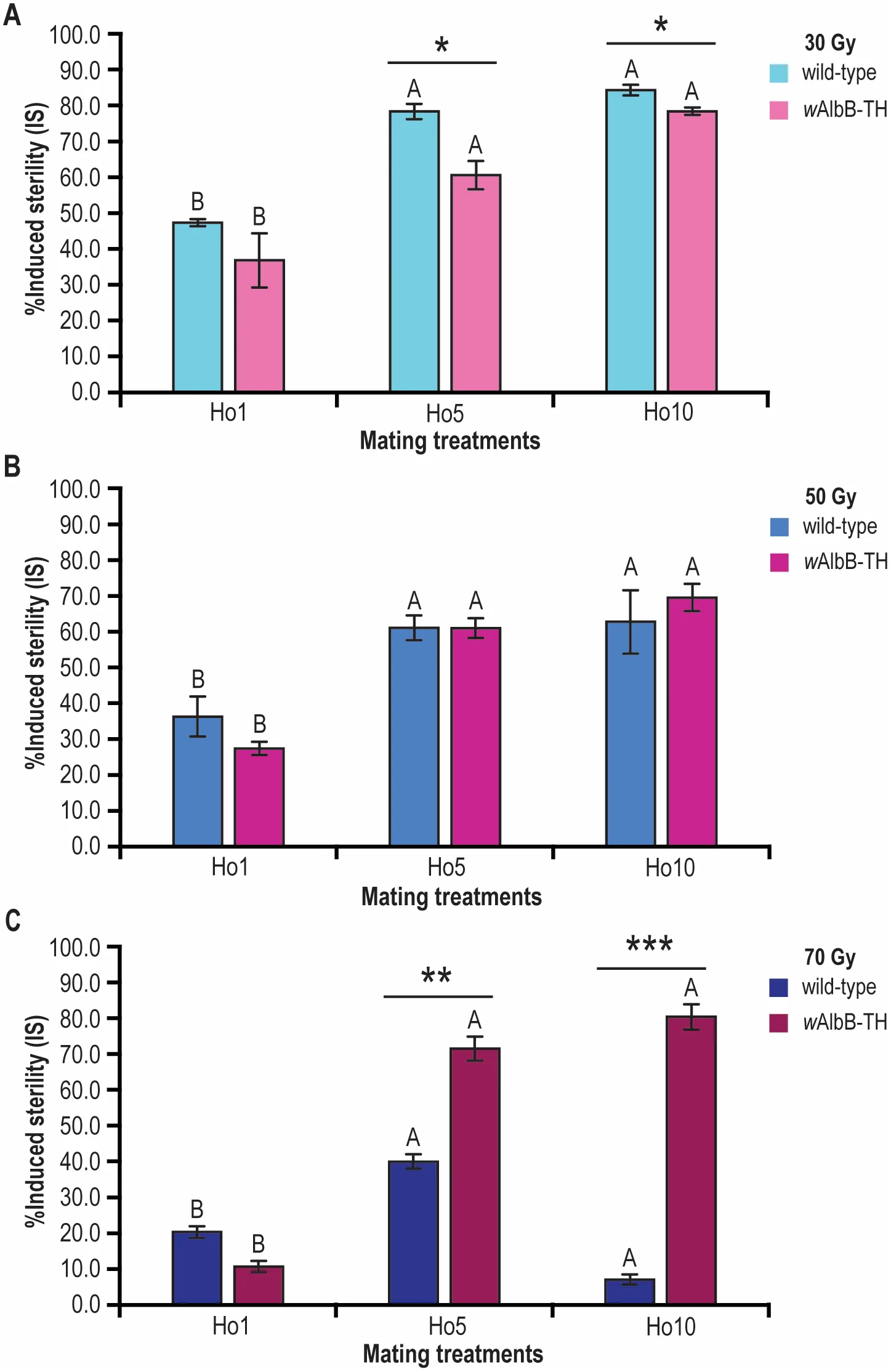
Induced sterility percentages in irradiated wild-type and *w*AlbB trans-infected *Aedes aegypt*i under different radiation doses: (A) 30 Gy, (B) 50 Gy, and (C) 70 Gy. Mating ratios were Ho1 (1:1), Ho5 (1:5), and Ho10 (1:10). Non-identical uppercase letters denote significant differences within wild-type treatments, and non-identical lowercase letters denote differences within *w*AlbB trans-infected treatments (*p* < 0.05). Asterisks indicate significant differences between mosquito colony (* for *p* < 0.05, ** for *p* < 0.01, *** for *p* < 0.001). Error bars represent SE from three replicates per treatment.

Induced sterility (IS) of 30 Gy-treated wild-type males was significantly higher than for the *w*AlbB trans-infected males in Ho5 and Ho10 (Ho1: Welch’s *t*-test, *p* = 0.298; Ho5: Independent *t*-test, *p* = 0.017; Ho10: Independent *t*-test, *p* = 0.031) (Fig 6A). At 70 Gy, *w*AlbB trans-infected males induced greater sterility than the wild type ones, particularly at Ho5 and Ho10 (Ho1: Independent *t*-test, *p* = 0.215; Ho5: Independent *t*-test, *p* = 0.001; Ho10: Independent *t*-test, *p* < 0.001) (Fig 6C). For 50 Gy, %IS did not differ significantly between wild-type and *w*AlbB trans-infected groups across observed ratios (Ho1: Independent *t*-*t*est, *p* = 0.202; Ho5: Independent *t*-*t*est, *p* = 0.971; Ho10: Independent *t*-*t*est, *p* = 0.518) (Fig 6B).

In wild-type males, 30 Gy produced the highest %IS across all mating ratios (Ho1: *F*_*2,6*_ = 7.626, *p* = 0.023, Ho5: *F*_*2,6*_ = 53.084, *p* < 0.001, Ho10: *F*_*2,6*_ = 57.761, *p* < 0.001) (S2A Fig). In *w*AlbB trans-infected males, 30 Gy induced significantly higher sterility than 70 Gy-irradiated treatments in Ho1 observed ratio (Kruskal-Wallis test, *df* = 2, *p* = 0.038) (S2B Fig). The induced sterility (%IS) was similar across treatments in Ho5 (60.6–71.5%, *F*_*2,6*_ = 3.329, *p* = 0.106) and Ho10 (78.4–80.3%, *F*_*2,6*_ = 3.635, *p* = 0.092) of *w*AlbB trans-infected group.

The %IS of irradiated wild-type groups was affected by both sterile male ratios and irradiation dosages, while the sterile male ratio had a stronger impact on the %IS of *w*AlbB trans-infected groups than the irradiation dosages.

Fried competitiveness index (C-value) of wild-type males at 50 Gy and 70 Gy treatments showed significantly lower competitiveness than 30 Gy at all mating ratios (Ho1: *F*_*2,6*_ = 16.771, *p* = 0.003; Ho5: Kruskal-Wallis test, *df* = 2, *p* = 0.027; Ho10: Kruskal-Wallis test, *df* = 2, *p* = 0.044) (Table 1). The competitiveness of wild-type males at 30 Gy increased with higher sterile male ratios, which contrasted with 50 Gy and 70 Gy results, where the competitiveness decreased at the highest ratios.

**Table 1:** Fried competitiveness index (C-value) of irradiated sterile wild-type and *w*AlbB trans-infected *Aedes aegypti* males under different doses (30 Gy, 50 Gy, 70 Gy) and male ratios (Ho1, Ho5, Ho10). Identical letters indicate no statistically significant difference among radiation doses (*p* > 0.05). *P*-value from *t*-test compares the C values between wild-type and *w*AlbB trans-infected males for each treatment. Asterisk indicates significant difference between colonies (*for *p* < 0.05). SE stands for standard error from three replicates.

Male mating competitive treatments		Fried competitiveness index (C-value) (Mean±SE) (95% C.I.)		*P*-value
**Sterile ratio**	**Radiation dosage (Gy)**	**wild-type** ***Ae. aegypti***	***w*AlbB-infected** ***Ae. aegypti***	
Ho1	30	1.12 ± 0.02 **A** (1.09–1.16)	0.64 ± 0.22 **a** (-0.32–1.58)	Welch’s *t*-test, *p* = 0.151
	50	0.60 ± 0.05 **B** (0.50–0.70)	0.38 ± 0.04 **ab** (0.23–0.53)	Independent *t*-test, *p* = 0.224
	70	0.27 ± 0.03 **B** (0.21–0.34)	0.12 ± 0.02 **b** (0.04–0.20)	Independent *t*-test, *p* = 0.196
Ho5	30	1.49 ± 0.09 **A** (1.30–1.67)	0.32 ± 0.05 **b** (0.10–0.53)	Welch’s *t*-test, *p* = 0.045*
	50	0.73 ± 0.02 **AB** (0.69–0.76)	0.72 ± 0.03 **a** (0.57–0.86)	Independent *t*-test, *p* = 0.873
	70	0.13 ± 0.00 **B** (0.13–0.14)	0.52 ± 0.08 **ab** (0.19–0.85)	Welch’s *t*-test, *p* = 0.033*
Ho10	30	1.92 ± 0.22 **A** (1.47–2.36)	0.37 ± 0.02 **a** (0.27–0.46)	Welch’s *t*-test, *p* = 0.140
	50	0.20 ± 0.03 **B** (0.15–0.26)	0.24 ± 0.04 **a** (0.08–0.40)	Independent *t*-test, *p* = 0.719
	70	0.08 ± 0.00 **B** (0.08–0.09)	0.44 ± 0.09 **a** (0.04–0.84)	Independent *t*-test, *p* = 0.021*

In *w*AlbB trans-infected males, the 30 Gy (C = 0.64 ± 0.22) had higher C-values than 50 Gy (C = 0.38 ± 0.04) and 70 Gy (C = 0.12 ± 0.02) under the Ho1 ratio (Kruskal-Wallis test, *df* = 2, *p* = 0.039). Although the C-values under the Ho5 ratio fluctuated, all radiation treatments of *w*AlbB trans-infected males showed no significant difference in C-values of the Ho10 observed ratios (*F*_*2,6*_ = 2.861, *p* = 0.134).

Both wild-type and *w*AlbB trans-infected males in 30 Gy treatments had the highest male competitiveness compared to other radiation dosages in the Ho1 treatment. High radiation reduced the competitiveness index in wild-type males across all observed ratios. The competitiveness index of 70 Gy-irradiated *w*AlbB trans-infected males was higher than 70 Gy-irradiated wild-type male groups in Ho5 and Ho10 observed ratios.

## Discussion

This study found that X-ray radiation between 30 Gy to70 Gy did not adversely affect adult emergence in wild-type *Ae. aegypti*, with 100% emergence observed in both sexes. Previous studies using gamma irradiation reported emergence rates ranging from 90.8% to 97.3% across 15 Gy to 90 Gy [16]. However, X-ray irradiation slightly reduced emergence in *w*AlbB trans-infected *Ae. aegypti*, with 92.7% to 98.7% in males and 88.0% to 96.0% in females, consistent with Axford et al. (2016) [41], who reported 97.83% to 98.92% emergence in non-irradiated *Wolbachia*-infected *Ae. aegypti* males. Notably, no significantly difference was observed in emergence between non-irradiated wild-type and *w*AlbB trans-infected mosquitoes. These findings suggest that *Wolbachia* infection became detrimental to emergence when combined with radiation exposure, particularly in females. Irradiation may disrupt host-microbe interactions, influencing emergence rates.

X-ray irradiation reduced mosquito survival, particularly at 50 Gy to 70 Gy, in both wild-type and *w*AlbB trans-infected mosquitoes*.* These results aligned with studies showing reduced longevity in *Ae. albopictus* irradiated with 60 Gy [33,42]. Radiation induced somatic damage, decreasing survival.

Interestingly, 30 Gy-irradiated *w*AlbB trans-infected mosquitoes showed greater longevity than non-irradiated controls, a contrast not seen in wild-type mosquitoes. This may reflect a hormetic effect, where low-dose radiation improves physiological resilience, consistent with prior studies. Calabrese et al. (2013) [43] reviewed the effects of radiation on insect longevity in *Drosophila*, tsetse fly, and *Ae. aegypti* mosquito and indicated that exposure to low radiation dosages at the early developmental stage of insects could enhance insect longevity [44–46]. Low X-ray irradiation dosages (30 Gy) may slow aging and extend life span in *w*AlbB trans-infected mosquitoes. However, the mechanism underlying the absence of this effect in wild-type mosquitoes remains unclear.

While adult emergence, survival, and longevity are crucial for sterile insect release, flight ability is also a key. Prior work has shown comparable flight ability between irradiated and non-irradiated mosquitoes [47], suggesting radiation primarily affects survival rate and longevity rather than flight ability.

In male sterility tests, fertility declined with increasing X-ray doses (S2 Table). At 30 Gy, sterility levels were similar between wild-type and *w*AlbB trans-infected males. At 50 Gy and 70 Gy, *w*AlbB trans-infected males were almost completely sterile, while wild-type males showed < 2% fertility. These results support prior findings showing near complete sterility in *Ae. aegypti* exposed to gamma radiation at 50 Gy to 120 Gy [48]. Gamma radiation appeared more effective at inducing sterility than X-ray [42]. Ionizing radiation damages mosquito cells and DNA, partially disrupting spermatogenesis and chromosome recombination, leading to the death of developing germ cells and reducing egg production [11,15,49]. It appeared that sterility depends on species, *Wolbachia* infection status, radiation source, and radiation dose.

Our study observed a reduction in relative *Wolbachia* density in whole-body *Ae. aegypti* samples following radiation treatment. This reduction occurred in both males and females across all age groups (days 7, 14, and 21) compared to non-irradiated controls. Similarly, Moretti et al., (2022) [15] reported lower *Wolbachia* loads in whole-body irradiated *Ae. albopictus* females on days 6 and day 13 compared to controls, attributing this decline primarily to ovarian damage. Zhang et al., (2023) [14] also demonstrated that X-ray-induced sterility in female *Ae. aegypti* results from the elimination of somatic supporting cells (including IGS and follicular cells), which blocks ovariole maturation; conversely, male sterility was linked to chromosomal damage in germ cells. As bacteria are generally less sensitive to radiation than eukaryotes [50] and *Wolbachia* residing concentratedly in the mosquito germline [51], irradiation may damage this niche or the bacteria themselves, resulting in reduced *Wolbachia* density.

Regarding age-dependence, we did not observe a clear trend in *Wolbachia* density for irradiated mosquitoes or non-irradiated females. A significant decrease was observed only in aged male mosquitoes at Day 21. In contrast, Calvitti et al. (2015) [52] reported that in non-irradiated *Ae. albopictus* naturally infected with both *w*AlbA and *w*AlbB of *Wolbachia*, only *w*AlbB density gradually increased, peaking at 16–20 days for males and 10–15 days for females*.* On the other hand, the density of *w*AlbA decreased over time in males, whereas it increased in females. Evidence suggests that lower *w*AlbA abundance in aged *Ae. albopictus* mosquitoes correlated with reduced CI efficiency [52,53]. Hence, it is important to monitor *Wolbachia* infection dynamics since early post-exposure in irradiated mosquitoes in the future study in order to assess the efficiency of control strategies.

The homothorax (*hth*) gene was selected as the host reference gene because it is an ultra-conserved single-copy gene widely used for normalization in mosquito qPCR assays [54,55]. To address this concern, we analyzed *hth* Ct values across all ages (day 7: 24.87 ± 1.04, day 14: 24.96 ± 0.88, day 21: 24.80 ± 0.56; *p* = 0.294). Using the mixed-effects model, the analysis revealed no significant effect of irradiation treatment on *hth* Ct values, indicating that host gene copy number remained stable across experimental conditions. Therefore, the observed changes in *Wolbachia* density are unlikely to be artifacts of normalization and instead reflect genuine biological effects.

Crosses between irradiated *w*AlbB trans-infected males and wild-type females (1:1 ratio) resulted in complete sterility, out-performing crosses involving irradiated wild-type or *w*AlbB trans-infected males mated with their respective female counterparts. This highlights the enhanced effectiveness of the combined SIT/IIT approach. High doses of X-ray irradiation induced stress on *Wolbachia* within the male reproductive organs, thereby reducing insect productivity [56]. Cytoplasmic incompatibility (CI) further contributed to sterility, compensating for any incomplete sterility from irradiation. CI is driven by *Wolbachia* protein that disrupts paternal chromosome segregation during anaphase of early mitosis, leading to embryo death [57]. Notably, despite the reduction in *Wolbachia* density, the CI trait remained functional. Thus, the combined SIT/IIT strategy offers an additional advantage over SIT alone by incorporating the sterility-induced effects of *Wolbachia-*mediated CI.

Notably, 30 Gy-irradiated *w*AlbB trans-infected males had lower C values than their wild-type counterparts (Table 1), consistent with previous reports of reduced sperm production in *Wolbachia*-infected males [56,58,59]. *Wolbachia* depleted seminal fluid proteins (Sfps) in semen modifying male spermatids that were transferred to *Drosophila melanogaster* females [60,61]. So far, decreasing Sfps in *w*AlbB trans-infected male semen might impair mating success. Further research is needed on *Wolbachia*-Sfps interaction in *Ae. aegypti*.

Kittayapong et al. (2025) [47] reported higher Fried index value for 50 Gy of gamma-irradiated males than found here with X-ray, again suggesting that higher efficacy of gamma rays. Complete sterility was achieved with gamma rays but not with X-rays at the same dose. *Ae. aegypti* were more sensitive to gamma radiation than to X-rays in terms of sterility, which corresponding to Wang et al. (2023) [42].

In male mating competitiveness experiment, multiple mating (polyandry) could potentially confound sterility efficiency results, particularly given that the mechanisms of mixed sperm usage within the spermathecae remain ambiguous [62]. Generally, *Ae. aegypti* females are considered monandrous, mating only once per lifetime [63], because seminal fluid proteins transferred during mating render them refractory to further copulation. Consequently, effective polyandry resulting in offspring with multiple fathers is extremely rare. Richardson et al. (2015) [64] demonstrated that only 6.25% of laboratory-reared females produced offspring with diverse paternal genetic backgrounds. More recent laboratory studies found that approximately 9% of females were inseminated with dual sperm types [62,65]. While re-mating was frequently attempted, 63% of second matings occurred almost exclusively within a short window (median 24 s; within 5 min) immediately following the first mating, of which 9% successful insemination [62]. Conversely, delayed second copulations (observed up to 96 h) consistently failed to result in spermathecal insemination. In semi-field enclosures, polyandry insemination rates were slightly higher at 14% [66]. This suggests that typical mating competitiveness tests may not perfectly represent free-ranging field behavior, particularly for irradiated mosquitoes. However, notably, sterile sperms appear to remain as competitive as fertile ones, capable of securing fertilization even in the cases of multiple inseminations or lower transfer quantities [62]. Therefore, our results should be interpreted within the context of these limitations.

The mating success probability shown in S3 Fig, derived from the histogram of larval hatching rates, serves as a proxy for female mating outcomes across various fertile-to-sterile male ratios under controlled conditions. In the fertile control, hatching rates are predominantly distributed in higher bins (0.9–1.0), whereas in the sterile control, hatching rates are concentrated in lower bins (0.0–0.1). As the sterile-to-fertile male ratios increased, the distribution shifted toward lower hatching rates, indicating the competitive mating success of sterile males. However, outliers in the probability of male mating success remain; these maybe partially attributed to polyandry. Consequently, our practical recommendations concerning irradiation dosage and release ratio require further validation under semi-field conditions prior to operational application.

Evaluation of key quality control parameters further supports the selection of 30 Gy as the most suitable irradiation dose for both wild-type and *w*AlbB trans-infected *Ae. aegypti*. Pupal mortality at 30 Gy was 0% in wild-type and 1.33% in *w*AlbB trans-infected male mosquitoes (S3 Table). The reduction in adult survival of 30 Gy-irradiated wild-type males was 2.78% relative to non-irradiated controls, whereas *w*AlbB trans-infected males showed higher survival compared to controls under the same conditions. At 30 Gy, both wild-type and *w*AlbB trans-infected male mosquitoes met key performance benchmarks under laboratory conditions, including low pupal mortality during irradiation and minimal reductions in adult survival (<10% relative to non-irradiated controls), consistent with IAEA quality control guidance [67].

Comparison of SIT and combined SIT/IIT approaches showed that irradiation at 30 Gy produced the highest induced sterility and C value across mating ratios in cage assays (Fig 6 and Table 1). At a 10:1 sterile-to-wild male ratio, induced sterility reached 84.27% in the wild-type group and 78.40% in the *w*AlbB-infected group. At a 1:1 ratio, Fried competitiveness indices were highest at 30 Gy (wild-type: C = 1.12; *w*AlbB-infected: C = 0.64), exceeding or approaching the IAEA-recommended threshold (C > 0.7). While the IAEA recommends induced sterility exceeding 99% for operational SIT programs, these guidelines also emphasize the importance of maintaining male quality and mating competitiveness; therefore, dose optimization under laboratory conditions necessarily involves evaluating sub-sterilizing doses to identify thresholds that maximize population-level suppression prior to semi-field or field implementation

On another hand, higher irradiation doses (50 Gy–70 Gy) resulted in decreased fertility percentage and were consistently associated with marked reductions in emergence, survival, and mating performance. A 10:1 sterile-to-wild male release ratio was identified as optimal, in agreement with previous field and semi-field studies [10].

Our findings suggested that male mating competitiveness plays a decisive role in population-level suppression and may outweigh the benefits of maximizing radiation-induced sterility alone. Notably, although 30 Gy induced only partial sterility in wild-type males, the superior competitiveness observed at this dose resulted in greater overall induced sterility at the population level compared with higher irradiation doses.

Taken together, these results demonstrate that 30 Gy represents the optimal compromise between biological efficacy and operational feasibility in laboratory-based evaluations. This supports its selection for subsequent semi-field and small-scale SIT and combined SIT/IIT programs prior to full-scale field applications.

In conclusion, increasing X-ray irradiation doses (30 Gy–70 Gy) progressively reduced adult emergence, survival, *Wolbachia* density, and male mating competitiveness, while cytoplasmic incompatibility remained unaffected. At 30 Gy, the lowest impact on adult survival was observed in both wild-type and *w*AlbB trans-infected *Ae. aegypti*. At this dose, both *w*AlbB-infected and wild-type *Ae. aegypti* males achieved effective sterility, as evidenced by reduced larval hatching rates relative to controls, while maintaining ecological fitness and mating competitiveness under laboratory conditions. Based on these laboratory and cage evaluations, a 30 Gy irradiation dose combined with a 10:1 sterile-to-fertile male release ratio appears to provide a suitable balance between biological performance and sterility induction under controlled conditions. However, further validation under semi-field and field conditions is required before operational application in SIT or combined SIT/IIT-based population suppression programs.

## Supporting information

S1 Table Adult longevity. (DOCX)

S2 Table Fertility percentage of male sterility test. (DOCX)

S3 Table Pupal mortality and relative adult survival. (DOCX)

S4 Table Data used to generate Fig 1. (DOCX)

S5 Table Data used to generate Fig 3. (DOCX)

S6 Table Data used to generate Fig 4. (DOCX)

S7 Table Data used to generate Fig 5. (DOCX)

S8 Table Data used to generate Fig 6. (DOCX)

S1 Fig Comparison of hatching rate under male mating competitiveness ratios. (DOCX)

S2 Fig Comparison of the induced sterility percentage between radiation dosages. (DOCX)

S3 Fig The probability of male mating success. (DOCX)

S4 Fig Experiment setting. (TIFF)

S1 Data Data used to generate Fig 2. (XLSX)

S2 Data Data used to generate Table 1. (XLSX)

S3 Data Data used to generate S3 Fig. (XLSX)
