# Supplementary material for: Effects of different X-ray irradiation dosages on Wolbachia trans-infected and uninfected Aedes aegypti mosquitoes
Source: PLoS Negl Trop Dis. 2026 Aug 28;20(8):e0014563. doi: 10.1371/journal.pntd.0014563 (PMC13524269; doi:10.1371/journal.pntd.0014563)
Supplement: Supplementary file 1 [file pntd.0014563.s001.docx]

**S1 Table.** Adult longevity

Adult longevity of wild-type *Aedes aegypti* (Aae-JJ) and *w*AlbB-infected *Ae. aegypti* (*w*AlbB-TH) was classified by sex and radiation dosages when pupae were irradiated with X-ray at 30 Gy, 50 Gy, and 70 Gy. (SE: Standard Error and CI: Confidence Interval)

|  | **Radiation**  **dosage (Gy)** | **Median survival time (Days) (95% C.I.)** | **Min longevity**  **(Days)** | **Max longevity**  **(Days)** |
| --- | --- | --- | --- | --- |
| ♂wild-type | 0 Gy | 36.0 (35–37) | 4 | 49 |
|  | 30 Gy | 35.0 (32–38) | 5 | 48 |
|  | 50 Gy | 23.0 (22–29) | 1 | 50 |
|  | 70 Gy | 19.0 (18–22) | 3 | 48 |
| ♀wild-type | 0 Gy | 39.0 (35–42) | 1 | 55 |
|  | 30 Gy | 25.0 (20–30) | 1 | 54 |
|  | 50 Gy | 29.0 (23–30) | 4 | 52 |
|  | 70 Gy | 14.0 (11–15) | 1 | 31 |
| ♂*w*AlbB-TH | 0 Gy | 22.0 (19–32) | 2 | 48 |
|  | 30 Gy | 36.0 (35–41) | 1 | 53 |
|  | 50 Gy | 20.0 (19–30) | 5 | 57 |
|  | 70 Gy | 20.0 (19–22) | 1 | 50 |
| ♂*w*AlbB-TH | 0 Gy | 20.0 (20–23) | 6 | 53 |
|  | 30 Gy | 30.5 (25–35) | 2 | 55 |
|  | 50 Gy | 27.5 (20–31) | 2 | 53 |
|  | 70 Gy | 19.0 (18–22) | 8 | 51 |
