# Supplementary material for: Effects of different X-ray irradiation dosages on Wolbachia trans-infected and uninfected Aedes aegypti mosquitoes
Source: PLoS Negl Trop Dis. 2026 Aug 28;20(8):e0014563. doi: 10.1371/journal.pntd.0014563 (PMC13524269; doi:10.1371/journal.pntd.0014563)
Supplement: Supplementary file 2 [file pntd.0014563.s002.docx]

**S2 Table.** Fertility percentage of male sterility test

Fertility percentages of wild-type and *w*AlbB trans-infected *Aedes aegypti* irradiated at different irradiation dosages of 30 Gy, 50 Gy, and 70 Gy, along with a non-irradiation control (0 Gy). Identical letters indicate no statistically significant differences among radiation doses (*p* > 0.05). *P*-values from Mann–Whitney U test compare fertility percentage between wild-type and *w*AlbB trans-infected males for each treatment. Asterisks indicate significant differences between colonies.

| **Radiation dosage (Gy)** | **Fertility percentage (Mean±SE)**  **95% C.I.** | | ***P*-value** |
| --- | --- | --- | --- |
|  | **Wild-type**  ***Ae. aegypti*** | ***w*AlbB-infected**  ***Ae. aegypti*** |  |
| 0 | 77.7±1.8 a | 63.7±2.9 a | < 0.001* |
| 30 | 11.9±1.5 b | 16.1±2.6 b | 0.522 |
| 50 | 1.9±0.5 c | 0.5±0.4 c | < 0.001* |
| 70 | 0.03±0.02 c | 0.00±0.00 c | 0.539 |
| * Significant difference when *p* < 0.05 | | | |
