# Supplementary material for: Effects of different X-ray irradiation dosages on Wolbachia trans-infected and uninfected Aedes aegypti mosquitoes
Source: PLoS Negl Trop Dis. 2026 Aug 28;20(8):e0014563. doi: 10.1371/journal.pntd.0014563 (PMC13524269; doi:10.1371/journal.pntd.0014563)
Supplement: Supplementary file 3 [file pntd.0014563.s003.docx]

**S3 Table.** Pupal mortality and relative adult survival

| **Mosquito colony** | **Radiation**  **dosage (Gy)** | **% Pupal mortality (Mean±SE)** | **Relative adult survival (%) compared to non-irradiated controls** |
| --- | --- | --- | --- |
| ♂wild-type | 0 Gy | 0 | - |
|  | 30 Gy | 0 | -2.78 |
|  | 50 Gy | 0 | -36.11 |
|  | 70 Gy | 0 | -47.22 |
| ♀wild-type | 0 Gy | 0 | - |
|  | 30 Gy | 0 | -35.90 |
|  | 50 Gy | 0 | -25.64 |
|  | 70 Gy | 0 | -64.10 |
| ♂*w*AlbB-TH | 0 Gy | 2.00±1.15 | - |
|  | 30 Gy | 1.33±0.33 | +63.64 |
|  | 50 Gy | 4.00±2.00 | -9.09 |
|  | 70 Gy | 7.33±2.60 | -9.09 |
| ♀*w*AlbB-TH | 0 Gy | 1.67±1.67 | - |
|  | 30 Gy | 4.00±1.15 | +52.50 |
|  | 50 Gy | 10.00±1.53 | +37.50 |
|  | 70 Gy | 12.00±1.15 | -5.00 |

“−” indicates reduced adult survival, and “+” indicates increased adult survival relative to the non-irradiated control.
