# Supplementary material for: Effects of different X-ray irradiation dosages on Wolbachia trans-infected and uninfected Aedes aegypti mosquitoes
Source: PLoS Negl Trop Dis. 2026 Aug 28;20(8):e0014563. doi: 10.1371/journal.pntd.0014563 (PMC13524269; doi:10.1371/journal.pntd.0014563)
Supplement: Supplementary file 4 [file pntd.0014563.s004.docx]

**S4 Table.** Data used to generate Figure 1

Adult emergence percentages of wild-type *Aedes aegypti* (Aae-JJ) and *w*AlbB-infected *Ae. aegypti* (*w*AlbB-TH) was classified by sex and radiation dosages when pupae were irradiated with X-ray at 30 Gy, 50 Gy, and 70 Gy. (SE: Standard Error and CI: Confidence Interval)

| **Mosquito**  **colony** | **Radiation dosage (Gy)** | **N** | **% Adult emergence**  **(Mean±SE)** | **95% CI** |
| --- | --- | --- | --- | --- |
| ♂︎wild-type | 0 (control) | 3 | 100.0±0.0 | 0.0 – 0.0 |
|  | 30 | 3 | 100.0±0.0 | 0.0 – 0.0 |
|  | 50 | 3 | 100.0±0.0 | 0.0 – 0.0 |
|  | 70 | 3 | 100.0±0.0 | 0.0 – 0.0 |
| ♀︎wild-type | 0 (control) | 3 | 100.0±0.0 | 0.0 – 0.0 |
|  | 30 | 3 | 100.0±0.0 | 0.0 – 0.0 |
|  | 50 | 3 | 100.0±0.0 | 0.0 – 0.0 |
|  | 70 | 3 | 100.0±0.0 | 0.0 – 0.0 |
| ♂︎ *w*AlbB-TH | 0 (control) | 3 | 98.0±1.2 | 95.4 – 100.6 |
|  | 30 | 3 | 98.7±0.3 | 96.0 – 101.3 |
|  | 50 | 3 | 96.0±2.0 | 93.4 – 98.6 |
|  | 70 | 3 | 92.7±2.6 | 90.1 – 95.3 |
| ♀︎ *w*AlbB-TH | 0 (control) | 3 | 98.3±1.7 | 96.2 – 100.4 |
|  | 30 | 3 | 96.0±1.2 | 93.9 – 98.1 |
|  | 50 | 3 | 90.0±1.5 | 87.9 – 92.1 |
|  | 70 | 3 | 88.0±1.2 | 85.9 – 90.1 |
