# Supplementary material for: Effects of different X-ray irradiation dosages on Wolbachia trans-infected and uninfected Aedes aegypti mosquitoes
Source: PLoS Negl Trop Dis. 2026 Aug 28;20(8):e0014563. doi: 10.1371/journal.pntd.0014563 (PMC13524269; doi:10.1371/journal.pntd.0014563)
Supplement: Supplementary file 5 [file pntd.0014563.s005.docx]

**S5 Table.** Data used to generate Figure 3

| **Male mating sterility treatments** | **N** | **Eggs (Mean±SE)**  **(95% CI)** | **Larvae (Mean±SE)**  **(95% CI)** |
| --- | --- | --- | --- |
| ♂︎ non-ir wild-type × ♀︎ non-ir wild-type | 92 | 77.34±3.57  (70.24–84.43) | 59.83±3.17  (53.54–66.12) |
| ♂︎ 30 Gy-ir wild-type × ♀︎ non-ir wild-type | 84 | 79.65±3.76  (72.18–87.13) | 9.08±0.95  (7.19–10.98) |
| ♂︎ 50 Gy-ir wild-type × ♀︎ non-ir wild-type | 97 | 75.33±3.85  (67.68–82.98) | 1.22±0.19  (0.84–1.59) |
| ♂︎ 70 Gy-ir wild-type × ♀︎ non-ir wild-type | 91 | 80.66±3.85  (73.01–88.31) | 0.02±0.02  (-0.01–0.05) |
| ♂︎ non-ir *w*AlbB-TH × ♀︎ non-ir *w*AlbB-TH | 68 | 75.43±3.50  (68.44–82.42) | 48.84±3.22  (42.41–55.27) |
| ♂︎ 30 Gy-ir *w*AlbB-TH × ♀︎ non-ir *w*AlbB-TH | 54 | 69.26±4.74  (59.75–78.77) | 12.50±2.57  (7.34–17.59) |
| ♂︎ 50 Gy-ir *w*AlbB-TH × ♀︎ non-ir *w*AlbB-TH | 42 | 61.62±4.49  (52.56–70.68) | 0.31±0.14  (0.03–0.59) |
| ♂︎ 70 Gy-ir *w*AlbB-TH × ♀︎ non-ir *w*AlbB-TH | 17 | 49.65±7.11  (34.57–64.72) | 0.00±0.00  (0.00–0.00) |
| ♂︎ non-ir A *w*AlbB-TH × ♀︎ non-ir wild-type | 84 | 78.17±3.34  (71.53–84.81) | 0.00±0.00  (0.00–0.00) |
| ♂︎ 30 Gy-ir *w*AlbB-TH × ♀︎ non-ir wild-type | 107 | 70.36±2.48  (65.45–75.27) | 0.00±0.00  (0.00–0.00) |
| ♂︎ 50 Gy-ir *w*AlbB-TH × ♀︎ non-ir wild-type | 100 | 71.17±3.13  (64.97–77.37) | 0.00±0.00  (0.00–0.00) |
| ♂︎ 70 Gy-ir *w*AlbB-TH × ♀︎ non-ir wild-type | 49 | 81.52±4.37  (72.73–90.31) | 0.00±0.00  (0.00–0.00) |
