# Supplementary material for: Effects of different X-ray irradiation dosages on Wolbachia trans-infected and uninfected Aedes aegypti mosquitoes
Source: PLoS Negl Trop Dis. 2026 Aug 28;20(8):e0014563. doi: 10.1371/journal.pntd.0014563 (PMC13524269; doi:10.1371/journal.pntd.0014563)
Supplement: Supplementary file 6 [file pntd.0014563.s006.docx]

**S6 Table.** Data used to generate Figure 4

*Wolbachia* density in males and females *w*AlbB trans-infected *Aedes aegypti*, classified by age and irradiation dosages of 30 Gy, 50 Gy, and 70 Gy. SE was the standard error from six biological replicates.

| **Radiation dosages** | **Age**  **(Days)** | ***Wolbachia* density (Mean±SE)**  **(95% C.I.)** | | | |
| --- | --- | --- | --- | --- | --- |
|  |  | **N** | **Male** | **N** | **Female** |
| 0  (non-irradiated) | 7 | 6 | 86.82±8.35  (67.26–110.21) | 6 | 81.20±17.41  (44.82–134.30) |
|  | 14 | 6 | 97.23±8.94  (76.30–122.27) | 6 | 98.13±52.27  (8.64–277.36) |
|  | 21 | 6 | 49.81±2.82  (42.95–57.46) | 6 | 72.09±11.95  (45.60–107.06) |
| 30 Gy | 7 | 6 | 23.26±2.93  (16.76–31.82) | 6 | 64.52±6.55  (49.47–83.12) |
|  | 14 | 6 | 48.50±2.09  (43.36–54.11) | 6 | 57.68±10.38  (34.77–88.12) |
|  | 21 | 6 | 48.11±3.24  (40.27–56.91) | 6 | 68.44±5.47  (55.39–83.53) |
| 50 Gy | 7 | 6 | 47.95±6.31  (33.53–65.95) | 6 | 50.50±9.53  (29.95–78.97) |
|  | 14 | 6 | 43.76±0.74  (41.89–45.70) | 6 | 82.42±18.92  (45.92–143.20) |
|  | 21 | 6 | 44.89±1.74  (40.60–49.53) | 6 | 49.69±2.61  (43.29–56.73) |
| 70 Gy | 7 | 6 | 28.77±2.24  (23.48–34.97) | 6 | 31.52±4.97  (20.49–46.06) |
|  | 14 | 6 | 28.41±0.37  (27.47–29.37) | 6 | 40.32±5.01  (28.92–54.68) |
|  | 21 | 6 | 40.32±4.60  (29.92–53.54) | 6 | 58.35±12.30  (32.64–95.90) |
