# Supplementary material for: Effects of different X-ray irradiation dosages on Wolbachia trans-infected and uninfected Aedes aegypti mosquitoes
Source: PLoS Negl Trop Dis. 2026 Aug 28;20(8):e0014563. doi: 10.1371/journal.pntd.0014563 (PMC13524269; doi:10.1371/journal.pntd.0014563)
Supplement: Supplementary file 7 [file pntd.0014563.s007.docx]

**S7 Table.** Data used to generate Figure 5

| **Male mating competitiveness treatments** | | **Hatch rate (Mean±SE)**  **(95% C.I.)** | | | |
| --- | --- | --- | --- | --- | --- |
| **Sterile**  **ratio** | **Radiation dosage (Gy)** | **N** | **Wild-type**  ***Ae. aegypti*** | **N** | ***w*AlbB-infected**  ***Ae. aegypti*** |
| Ho1 | 30 | 3 | 0.48±0.01  (0.44–0.52) | 3 | 0.59±0.07  (0.29–0.90) |
|  | 50 | 3 | 0.58±0.05  (0.36–0.80) | 3 | 0.68±0.02  (0.61–0.76) |
|  | 70 | 3 | 0.72±0.06  (0.48–0.97) | 3 | 0.84±0.02  (0.78–0.90) |
| Ho5 | 30 | 3 | 0.20±0.02  (0.11–0.28) | 3 | 0.37±0.04  (0.21–0.53) |
|  | 50 | 3 | 0.35±0.03  (0.22–0.49) | 3 | 0.37±0.03  (0.26–0.48) |
|  | 70 | 3 | 0.55±0.02  (0.47–0.63) | 3 | 0.27±0.03  (0.13–0.40) |
| Ho10 | 30 | 3 | 0.14±0.01  (0.08–0.20) | 3 | 0.20±0.01  (0.16–0.24) |
|  | 50 | 3 | 0.34±0.14  (-0.01–0.69) | 3 | 0.29±0.04  (0.13–0.44) |
|  | 70 | 3 | 0.50±0.03  (0.37–0.63) | 3 | 0.18±0.03  (0.04–0.33) |
