# Supplementary material for: Effects of different X-ray irradiation dosages on Wolbachia trans-infected and uninfected Aedes aegypti mosquitoes
Source: PLoS Negl Trop Dis. 2026 Aug 28;20(8):e0014563. doi: 10.1371/journal.pntd.0014563 (PMC13524269; doi:10.1371/journal.pntd.0014563)
Supplement: Supplementary file 8 [file pntd.0014563.s008.docx]

**S8 Table.** Data used to generate Figure 6

| **Male mating competitiveness treatments** | | **% Induced sterility (Mean±SE)**  **(95% C.I.)** | | | |
| --- | --- | --- | --- | --- | --- |
| **Sterile**  **ratio** | **Radiation dosage (Gy)** | **N** | **Wild-type**  ***Ae. aegypti*** | **N** | ***w*AlbB-infected**  ***Ae. aegypti*** |
| Ho1 | 30 | 3 | 47.35±1.00  (43.06–51.65) | 3 | 36.80±7.60  (4.09–69.50) |
|  | 50 | 3 | 36.32±5.57  (12.37–60.27) | 3 | 27.41±1.80  (19.64–35.17) |
|  | 70 | 3 | 20.33±6.37  (-7.09–47.76) | 3 | 10.66±1.60  (3.79–17.53) |
| Ho5 | 30 | 3 | 78.35±2.12  (69.21–87.49) | 3 | 60.60±3.94  (43.63–77.56) |
|  | 50 | 3 | 61.12±3.48  (46.16–76.08) | 3 | 60.95±2.76  (49.05–72.84) |
|  | 70 | 3 | 39.99±2.06  (31.12–48.86) | 3 | 71.48±3.36  (57.02–85.94) |
| Ho10 | 30 | 3 | 84.27±1.49  (77.86–90.69) | 3 | 78.40±1.02  (74.03–82.77) |
|  | 50 | 3 | 62.74±8.85  (24.69–100.80) | 3 | 69.54±3.77  (53.32–85.76) |
|  | 70 | 3 | 7.06±1.42  (0.97–13.15) | 3 | 80.34±3.48  (65.37–95.32) |
