# Supplementary material for: Effects of different X-ray irradiation dosages on Wolbachia trans-infected and uninfected Aedes aegypti mosquitoes
Source: PLoS Negl Trop Dis. 2026 Aug 28;20(8):e0014563. doi: 10.1371/journal.pntd.0014563 (PMC13524269; doi:10.1371/journal.pntd.0014563)
Supplement: Supplementary file 9 [file pntd.0014563.s009.docx]

**S1 Fig.** Comparison of the hatch rate under male mating competitiveness ratios

Egg hatch rates of wild-type and *w*AlbB trans-infected *Aedes aegypti* mated under male mating competitiveness ratios of (A) 30 Gy, (B) 50 Gy and (C) 70 Gy. Observed mating ratios were Ho1(1:1), Ho5(1:5), and Ho10 (1:10). Identical bars indicate no significant difference (*p* > 0.05). Asterisks represent *t*-test significance between wild-type and *w*AlbB trans-infected *Ae. aegypti* (* for *p* < 0.05*, and ** for *p* < 0.01). Error bars represent SE from three replicates.
