# Supplementary material for: Effects of different X-ray irradiation dosages on Wolbachia trans-infected and uninfected Aedes aegypti mosquitoes
Source: PLoS Negl Trop Dis. 2026 Aug 28;20(8):e0014563. doi: 10.1371/journal.pntd.0014563 (PMC13524269; doi:10.1371/journal.pntd.0014563)
Supplement: Supplementary file 10 [file pntd.0014563.s010.docx]

**S2 Fig.** Comparison of the induced sterility percentage between radiation dosages

Induced sterility percentage of wild-type (A) and *w*AlbB trans-infected (B) *Aedes aegypti* under observed mating ratios (Ho1, Ho5, and Ho10) after irradiation at 30 Gy, 50 Gy, and 70 Gy. Bars with identical or no letters denote no significant difference among radiation doses in each treatment (*p* > 0.05). Error bars indicate SE from three replicates.
