# Supplementary material for: Effects of different X-ray irradiation dosages on Wolbachia trans-infected and uninfected Aedes aegypti mosquitoes
Source: PLoS Negl Trop Dis. 2026 Aug 28;20(8):e0014563. doi: 10.1371/journal.pntd.0014563 (PMC13524269; doi:10.1371/journal.pntd.0014563)
Supplement: Supplementary file 11 [file pntd.0014563.s011.docx]

**S3 Fig.** The probability of male mating success

The histogram illustrates the probability of male mating success for irradiated sterile wild-type (A-C) and *w*AlbB trans-infected (D-F) *Aedes aegypti* males under different irradiation doses (30, 50, and 70 Gy). Control groups included Hn (only non-irradiated wild-type males) and Hs (only irradiated sterile males). Observed mating competitiveness was assessed at different male ratios between fertile and sterile males: Ho1 (1:1), Ho5 (1:5), and Ho10 (1:10). The probability of male mating success was inferred from larval hatching rates, calculated from eggs laid by individual females in each treatment. Hatching rates from three biological replicates per treatment were grouped into bins of 0.1 within the range of 0 to 1 for histogram construction. Below each histogram plot is a horizontal bee-swarming plot of larval hatching rates, calculated from eggs laid by individual females from the three replications. The red bar shows the median of larval hatching rate.
